# Individualized Clinical Practice Guidelines for Pressure Injury Management: Development of an Integrated Multi-Modal Biomedical Information Resource

**DOI:** 10.2196/10871

**Published:** 2018-09-06

**Authors:** Kath M Bogie, Guo-Qiang Zhang, Steven K Roggenkamp, Ningzhou Zeng, Jacinta Seton, Shiqiang Tao, Arielle L Bloostein, Jiayang Sun

**Affiliations:** ^1^ Louis Stokes Cleveland VA Medical Center Cleveland, OH United States; ^2^ Department of Orthopaedics Case Western Reserve University Cleveland, OH United States; ^3^ Institute for Biomedical Informatics University of Kentucky Lexington, KY United States; ^4^ Department of Computer Science University of Kentucky Lexington, KY United States; ^5^ Department of Population and Quantitative Health Sciences Case Western Reserve University Cleveland, OH United States

**Keywords:** bioinformatics, electronic health record, pressure injury

## Abstract

**Background:**

Pressure ulcers (PU) and deep tissue injuries (DTI), collectively known as pressure injuries are serious complications causing staggering costs and human suffering with over 200 reported risk factors from many domains. Primary pressure injury prevention seeks to prevent the first incidence, while secondary PU/DTI prevention aims to decrease chronic recurrence. Clinical practice guidelines (CPG) combine evidence-based practice and expert opinion to aid clinicians in the goal of achieving best practices for primary and secondary prevention. The correction of all risk factors can be both overwhelming and impractical to implement in clinical practice. There is a need to develop practical clinical tools to prioritize the multiple recommendations of CPG, but there is limited guidance on how to prioritize based on individual cases. Bioinformatics platforms enable data management to support clinical decision support and user-interface development for complex clinical challenges such as pressure injury prevention care planning.

**Objective:**

The central hypothesis of the study is that the individual’s risk factor profile can provide the basis for adaptive, personalized care planning for PU prevention based on CPG prioritization. The study objective is to develop the Spinal Cord Injury Pressure Ulcer and Deep Tissue Injury (SCIPUD+) Resource to support personalized care planning for primary and secondary PU/DTI prevention.

**Methods:**

The study is employing a retrospective electronic health record (EHR) chart review of over 75 factors known to be relevant for pressure injury risk in individuals with a spinal cord injury (SCI) and routinely recorded in the EHR. We also perform tissue health assessments of a selected sub-group. A systems approach is being used to develop and validate the SCIPUD+ Resource incorporating the many risk factor domains associated with PU/DTI primary and secondary prevention, ranging from the individual’s environment to local tissue health. Our multiscale approach will leverage the strength of bioinformatics applied to an established national EHR system. A comprehensive model is being used to relate the primary outcome of interest (PU/DTI development) with over 75 PU/DTI risk factors using a retrospective chart review of 5000 individuals selected from the study cohort of more than 36,000 persons with SCI. A Spinal Cord Injury Pressure Ulcer and Deep Tissue Injury Ontology (SCIPUDO) is being developed to enable robust text-mining for data extraction from free-form notes.

**Results:**

The results from this study are pending.

**Conclusions:**

PU/DTI remains a highly significant source of morbidity for individuals with SCI. Personalized interactive care plans may decrease both initial PU formation and readmission rates for high-risk individuals. The project is using established EHR data to build a comprehensive, structured model of environmental, social and clinical pressure injury risk factors. The comprehensive SCIPUD+ health care tool will be used to relate the primary outcome of interest (pressure injury development) with covariates including environmental, social, clinical, personal and tissue health profiles as well as possible interactions among some of these covariates. The study will result in a validated tool for personalized implementation of CPG recommendations and has great potential to change the standard of care for PrI clinical practice by enabling clinicians to provide personalized application of CPG priorities tailored to the needs of each at-risk individual with SCI.

**Registered Report Identifier:**

RR1-10.2196/10871

## Introduction

This project studies the development, validation, and timing of promising interventions to address consequences of spinal cord injury (SCI), specifically the primary and secondary prevention of pressure ulcers and deep tissue injury (PU/DTI), collectively known as pressure injuries (PrI). These chronic wounds are a major negative consequence of SCI. The Spinal Cord Injury Pressure Ulcer and Deep Tissue Injury (SCIPUD+) health care tool enables personalized PrI care planning, supporting identification and validation of best practices in SCI care for musculoskeletal health, and rehabilitation interventions.

More than 200 risk factors for PrI development have been reported for individuals with SCI [1], spanning multiple domains [2]. The Center for Medicare and Medicaid Services has determined that severe (ie, stage 3 and 4), hospital acquired PrI are entirely preventable “never events” and have discontinued reimbursement [3]. The clinical reality is that many people living with SCI continue to develop significant PrI, both in the community and hospital. Patients in acute care hospitals have 33% PrI incidence rates, with prevalence rates up to 69% [4,5]. On admission to skilled nursing facilities, PrI prevalence ranges between 10% and 26% [6,7]. Veterans with chronic SCI have incidence rates as high as 62% to 80% [8,9], and over their lifetime 34% will require at least three PrI related hospitalizations for treatment [10]. PrIs may lead to other serious medical complications, such as osteomyelitis, sepsis, and even death. In addition to the personal distress and negative impact on the quality of life (QoL) for the individual, PrI place a major cost burden on health care systems. PrI prevention is approximately 2.5 times more economical than treatment [11], with direct treatment costs for one stage 4 PrI exceeding US $100,000 over 6 years ago [12-15].

Primary PrI prevention is the first line of defense [16]. Clinical practice guidelines (CPGs) developed to aid clinicians in this goal combine a balance of evidence-based practice and expert opinion. There are multiple CPGs for PrI prevention [17-21], each with similar recommendations regarding risk assessment, prevention, PrI assessment, measurement, treatment and documentation. However, they also contain significant differences. The primary challenge with all CPGs is that there are many factors to consider. For example, the CPG from the Consortium for Spinal Cord Medicine, released in September 2014, contains a summary of over 25 recommendations to be followed by care providers [21]. Moreover, there is limited guidance on how to prioritize the recommendations for individual cases. It is overwhelming and even unrealistic to expect every recommendation to be implemented concurrently [2]. The relative importance of risk factors has not yet been investigated, limiting care planning, and prioritization of interventions. Unfortunately, as Thomason et al [22] found, although SCI physicians and nurses generally agree with the written CPG recommendations, they do not believe that these recommendations were fully implemented in their respective clinical settings. Furthermore, a European Pressure Ulcer Advisory Panel survey of PrI prevalence in 5000 hospitalized patients throughout Europe indicated that clinical expertise and standard treatment guidelines are not sufficient [23]. The International Pressure Ulcer Prevalence Study, conducted from 2006 to 2009, demonstrated an increase in PrI prevalence in the US. While overall PrI prevalence decreased modestly, the prevalence of suspected deep tissue injury increased during the same period [24]. The continued high incidence of chronic PrI, including recurrent wounds, underscores the need to develop new approaches to primary and secondary prevention.

The future of scientific research and evidence-based personalized practice will increasingly require multidisciplinary teams as the problems become more complex and the investigative tools more sophisticated. The Wound Healing Research Unit at Cardiff University, Wales initiated a multidisciplinary wound management team over 20 years ago [25]. This approach can optimize effective translation and validation of best practices for standard clinical practice. In 2013, the Veterans Health Administration (VHA) launched a 5-year strategic plan with the goal of moving the health care system for Veterans towards Personalized, Proactive, Patient-driven Care, delivered across the life continuum from prevention through tertiary care and end of life [26]. To achieve this goal for successful PrI management the patient-centered multidisciplinary team typically includes physicians, nurses, physical therapists, occupational therapists, dieticians, psychologists, and biomedical engineers [27,28].

Current PrI screening tools include a variety of risk assessment scales [9,29,30]. It is essential that they be validated as reliable for use within specific patient populations [31]. While sensitivity and specificity vary widely between scales, the Braden scale has the best balance for general population use (57.1%/67.5%) [32]. However, a review of the seven most widely used scales revealed that validation for use in the SCI population was limited [31], and there was a lack of reliability or responsiveness evidence for these individuals. A comparative effectiveness review of PrI risk assessment by the Agency for Health Care Research and Quality found no difference between clinical judgment and the use of scales [33]. Tescher et al [34] commented that all at-risk patients are not created equal and concluded that the Braden scale does not assist the clinician in developing individualized prevention plans. As noted by Pancorbo-Hidalog et al and others [32,35,36], there is no data to suggest that the use of risk assessment scales prevents PrI. Thus, it appears that the evidence regarding the effectiveness of risk-assessment tools for preventing PrI is insufficient.

Primary prevention of PrI incidence and secondary prevention of PrI recurrence depend on reliably identifying the risk factors that contribute to its formation. A multidisciplinary expert panel found that while PrI development is influenced by multiple variables, and many risk factors have already been identified, several critical questions remain unanswered and require further research [2]. Most of the published research on PrI risk focuses on either nursing home residents or the population with acute SCI. However, the degree to which these risk factors apply to other populations has not been established. PrI environmental risk factors may vary between rural and urban populations due to ease of access to transportation, access to specialized clinical care, and air quality. For example, the Veterans Affairs SCI population includes a high proportion of individuals who receive life-long care in both urban and rural areas, and who may have different rates of primary PrI development [37,38].

The continued high incidence of PrI for many individuals at-risk in the hospital and community indicates that CPG, standardized pressure relief regimes, and risk assessment scales alone are insufficient. PrI management remains complex and multidimensional. Motivational interviewing helps individuals to adhere to personal care plans [39,40]. However, focusing primarily on motivation using a standardized approach for individuals with SCI is ineffective for secondary prevention [34]. This highlights the continued need for a personalized approach.

Individuals with SCI are at increased risk of PrI development. However, this devastating consequence of SCI appears to be unique for everyone. A regime of regular postural alteration and pressure relief is considered essential to minimize the risk of PrI development. Still, some individuals remain PrI free without regular pressure relief, while others perform regular pressure relief and repeatedly develop tissue breakdown. The transition from the inpatient hospital or living in a nursing home to the community following rehabilitation may impact environmental risk factors. Likewise, living alone or with a partner can impact social risk factors. CPG consider all these factors but do not provide relative prioritization.

The correction of all PrI risk factors for an individual with SCI can be both overwhelming and impractical to implement in clinical practice. The need to develop effective clinical tools to prioritize the multiple recommendations of CPG has been identified by experts in the field. In preparative work, our development and application of the preliminary SCIPUD+ Resource has shown that risk factors for primary prevention may not be the same as those for secondary prevention (ie, PrI recurrence) [41].

**Figure 1:**
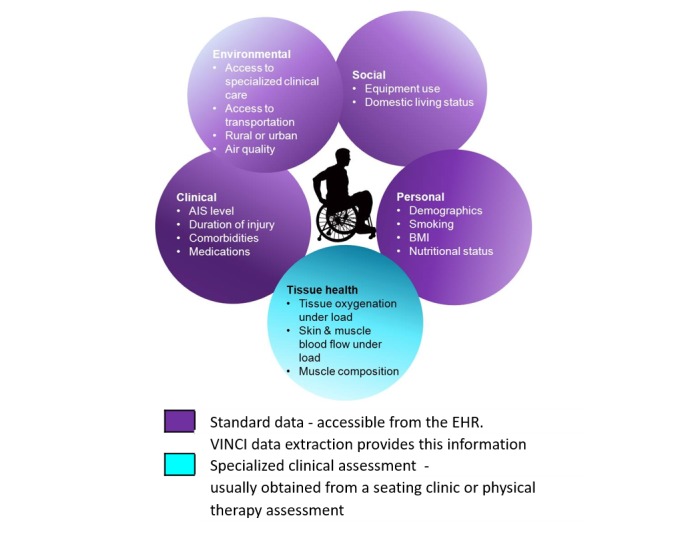
Multiple risk factor domains contribute to pressure ulcer (PU/DTI) risk. AIS: American Spinal Injury Association Impairment Scale; BMI: body mass index; EHR: electronic health record; VINCI: Veterans Affairs Informatics and Computing Infrastructure Database.

The application of biomedical informatics approaches enables systematic data extraction, storage, and analysis to provide clinical decision support and user-interfaces for addressing complex clinical challenges such as PrI prevention care planning. A systems approach is being used to develop and validate the SCIPUD+ Resource, a multivariate structural model that includes all core National Institute of Neurological Disorders and Stroke Common Data Elements (NINDS CDE) [42] and contributions from the many risk factor domains associated with PrI (Figure 1). These range from the individual’s environment to local tissue health. The goal of the SCIPUD+ Resource is to provide a personalized health care tool to address a major consequence of SCI, specifically PrI prevention care planning. Personalized interactive programs can enhance best practices in SCI care by decreasing both initial PrI formation and readmission rates due to PrI recurrence for high-risk individuals, particularly Veterans with SCI.

The objective of this study is to develop a structural model of environmental, social, and clinical factors to provide weighted systemic insight into PrI risk in people with SCI to support personalized care plans for primary and secondary PrI prevention. The SCIPUD+ Resource will be developed using data sets extracted from the Veterans Affairs Informatics and Computing Infrastructure (VINCI) database [43] together with a cross-sectional study of tissue health profiles. This will be validated using an observational cohort study. The central hypothesis of this study is that the individual’s risk factor profile provides the basis for adaptive, personalized care planning for PrI prevention based on CPG prioritization.

## Methods

### Study Design

This study employed a retrospective electronic health record (EHR) chart review of over 75 factors known to be relevant for PrI risk in individuals with SCI and routinely recorded in the EHR. We also perform tissue health assessments for a selected sub-group. Regulatory approval for the study was obtained from the local institutional review board. By applying a data-centric approach, we can leverage the power of the data resource provided by VINCI and the detailed personal characteristic database of tissue health to provide the weighted, adaptive, personalized SCIPUD+ Resource for primary and secondary PrI prevention.

The integrated SCIPUD+ Resource is being assembled from 2 databases: (1) one using data extracted from the VINCI EHR by informatics and text mining, and (2) another using tissue health data. PrI risk factor data collected at multiple retrospective time points include modifiable and nonmodifiable factors identified in cross-sectional and observational studies. Multiscale data extraction includes numerical, categorical and text data mining. A Spinal Cord Injury Pressure Ulcer and Deep Tissue Injury Ontology (SCIPUDO), is being developed to ensure robust and extensive information extraction from the free text clinical note. We will also carry out a cross-sectional study of tissue health profiles in a representative cohort of 60 individuals with SCI.

The Multi-Modality, Multi-Resource Information Integration Environment for Multi-Center Physiological and Clinical Research Studies (Physio-MIMI) cloud-based multi-modal data storage and access platform [44] creates a common Web-based user interface for data queries. It also enables the development of compatible analytical tools and easier sharing of complex data from multiple domains to support collaborative clinical and translational research using diverse data types. Another tool, Ontology-driven Web-based Research Data Capture (OnWARD) provides robust flexibility of input data storage in a relational database for detailed analysis. It can be quickly deployed and customized for any clinical study. OnWARD has eased the data entry burden in multiple clinical trials [45].

Structural modeling of factors from multiple domains and their co-impact on developing PrI will be used to provide weighted systemic insight into initial and recurrent PrI risk in people with SCI. A comprehensive model will be used to relate the primary outcome of interest (ie, PrI development) with covariates including environmental, social, clinical, personal, and tissue health profiles and possible interactions among some of these covariates.

### Cohort Extraction

The SCIPUD+ Resource is being developed using a detailed chart review of VINCI data employing International Classification of Diseases, Ninth Revision, Clinical Modification (ICD-9-CM) codes for paraplegia and tetraplegia with a secondary filter using an SCI-specific stop code. The search timeframe is the preconversion date (September 2010 to September 2015) because the International Classification of Diseases, Tenth Revision, Clinical Modification (ICD-10-CM) codes do not currently provide accurate delineation of SCI factors. The initial query returned approximately 36,000 different individuals and 120,000 encounters during the search timeframe across the VHA nationally. It was clarified that some individuals coded for SCI have a primary diagnosis of multiple sclerosis (MS) or amyotrophic lateral sclerosis (ALS). Therefore, we revised the code to develop a secondary filter to exclude individuals with MS and ALS since risk factors vary considerably in these neurodegenerative diseases compared to SCI. This secondary query revealed a study cohort of over 20,000 individuals with SCI, equivalent to about 8% of the total United States population. Within this cohort, a detailed review found that it includes more than 109,000 encounters. Furthermore, we have learned that each encounter encompasses an episode of care and may include different appointments stemming from the same visit, or an extended period of hospitalization. Thus, we have estimated the cohort includes about 500,000 different events and over 40 million data points.

**Figure 2:**
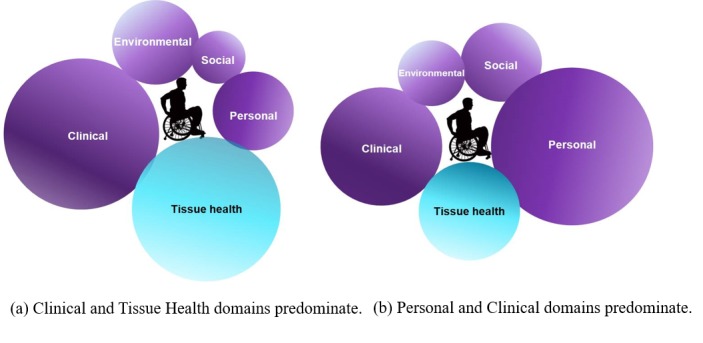
SCIPUD+ pressure ulcers/deep tissue injuries care plan model. Domain size indicates the relative importance for that individual.

Our research strategy builds on our existing methodologies to create the SCIPUD+ Resource to enable personalized care planning for PrI prevention based on the individual’s holistic characteristics [41]. Analysis of multiple PrI risk factors requires a robust and scalable informatics approach to cope with challenges in volume and complexity. Clinical and demographic data is collected using systems with a variety of sampling rates and formats. Even when checklists and coding are required, data may be missing or only found in the free form note. During preliminary work, we found that ICD-9-CM codes markedly under-reported the number of PrI treated. In a population of 399 eligible patients, only 93 (23.3%) were coded for PrI. We have developed a pathway for construction of disease-specific ontologies for data extraction using Natural Language Processing (NLP) for complex, specialized clinical notes. We will create the dedicated domain ontology SCIPUDO by reusing terminology from existing systems ranging from anatomy (Systematized Nomenclature of Medicine-Clinical Trials), disease classification (ICD-9-CMand ICD-10-CM), medication (website RxNorm for clinical trial drug standardized nomenclature), and NINDS CDE. Due to Physio-MIMI’s highly adaptable system architecture with domain ontology as a plug-and-play component, the proposed SCIPUD+ Resource can be developed by reusing much of the existing open-source tools that we have already developed. Figure 2 shows 2 hypothetical examples. In the first scenario, the clinical profile and tissue health response are the most critical domains. Potentially modifiable factors in these domains include spasticity and applied loads. Thus, the SCIPUD+ care plan would prioritize spasticity management and equipment provided. In the second scenario, the critical domains are personal and clinical factors. In addition to the potentially modifiable factors in the clinical domain, potentially modifiable factors in the personal domain include smoking and body mass index.

### Sample Size Calculation

Based on our prior data, we defined the expected PrI incidence as 30% and a clinically significant difference as reducing the incidence by 50%. The basic PrI status extracted from the EHR is PrI or not PrI, leading to a dichotomous outcome. However, the severity of PrI differs. We will use text mining to further classify wound status as severe PrI (stage 3 or 4), minor PrI (stage 1 or 2), deep tissue injury, absent or unclassified, leading to a polytomous outcome. A first-line analysis model for dichotomous outcome uses logistic regression, while the first-line analysis model for a polytomous outcome uses multinomial logistic regression. Considering all variables and their possible interactions would lead to approximately 3082 covariates to be studied in each model. In practice, it is reasonable to expect that only a small portion of these covariates, possibly as few as 25 would be enough to predict PrI outcomes. Only clinically meaningful interactions need to be considered at the start of our modeling. To achieve a reasonably rich SCIPUD+ database that allows for a balanced cohort selection of the personal, environmental, social, and clinical factors and for an extensive study of the impact of these factors, we can and will oversample. Therefore, data will be extracted from a retrospective chart review of 5000 individuals selected from the study cohort of over 36,000 individuals with SCI using a stratified sampling scheme. We will retain 500 representative cases for further validation and testing. This sampling will provide more than 1418 (ie, 5000 minus 3082 minus 500) degrees of freedom, which is more than enough to determine the top 25 predictors. It will also validate and test these predictors with at least 80% power under a standard significance level of 0.05, assuming an average difference of PrI incidence of at least 0.15, and a moderately balanced number of cases [46-48]. The validation and testing of these top 25 predictors will be based on standard tests and bootstrap procedures.

### Data Extraction and Processing

Raw data is extracted from VINCI using a stratified ICD-9-CM code search, de-identified and stored as a comma-separated values (CSV) file. Data formats include categorical, numerical and free-form text in clinical notes. Data to be collected includes factors identified as being possibly related to PrI development or healing either in cross-sectional or other observational studies. Annual evaluations for the complete study cohort will be included in the SCIPUD+ database, to determine changes over time. Any patient admission will also be reviewed, together with weekly in-patient and discharge encounters. We are particularly interested in the possible differences in PrI risk based on the level and extent of spinal injury and motor and sensory impairment. Thus, we will examine quadriplegia motor-complete (QMC), quadriplegia motor-incomplete (QMI), paraplegia motor-complete (PMC), and paraplegia motor-incomplete (PMI).

We are developing SCIPUDO as the knowledge resource for processing specialized terms related to SCI, PrI, and deep tissue injuries. Parsing and analyzing clinical narratives present a unique set of challenges that distinguish it from the broader biomedical NLP approaches. There has been extensive work in creating clinical NLP systems focused on information extraction from free text in specific disease domains, such as cancer [49] and tuberculosis [50]. However, there is no community gold standard for SCI or PrI named entities to date. Thus, we will build our gold standard using manual annotations created by clinical team members who will review a random sample of records from over 20,000 clinical notes for this cohort extracted from VINCI. One to 3 clinicians will review each record. The SCIPUDO will enable: (1) term disambiguation (ie, between commonly used synonyms and acronyms of a term such as *quadriplegia and tetraplegia*), (2) term normalization (ie, syntactic variations of a term, such as singular or plural and acronyms will be normalized using classes and customized rules such as *pressure ulcer*, *PU*, or *PrU*), and (3) subsumption reasoning using class hierarchy to allow terms to be classified according to their broader semantic type.

Validated extracted data will then be collated using our established standard data collection forms and uploaded to the Physio-MIMI based integrated PrI risk assessment SCIPUD+ Resource. The Physio-MIMI backbone will provide extensible, scalable, and high-performance data management for storing and accessing large volumes of data rapidly. Reliable data storage through automated data replication and data integrity verification will ensure consistent data availability and effective disaster recovery with off-site data backup. Data quality assurance and metadata version control will be managed using a combination of GitHub, JSON, and the open source NSR data management environment [51].

### Creation of the SCIPUD+ Environmental, Social and Clinical Domain Database

Input data for the SCIPUD+ database will be provided by synthesizing available EHR clinical data from VINCI, using a protocol based on our preliminary work. VINCI provides EHR data storage for all health care encounters within the VHA and is updated daily. A preliminary query on June 10, 2014, found that between 2009-2014 there were 16,076 individuals seen by the VHA with an ICD-9-CM code of 344.00 (ie, quadriplegia) and 24,052 individuals with an ICD-9-CM code of 344.1 (ie, paraplegia). Of these, 6420 (16.00%) in both groups were also coded for a PrI (ICD-9-CM code 707.00). Within the local area, there were 1021 encounters with individuals with quadriplegia and 1443 with paraplegia. The reported rate of PrI incidence was 14.00%. Extraction of clinical details will entail text mining of the free text clinical notes. SCIPUDO will enable robust text mining for data extraction from free form notes in addition to using ICD-9-CM codes to retrieve data of interest. A visual query interface will be adapted from OnWARD to allow all clinicians to directly query the SCIPUD+ Resource via a set of readily usable visual widgets that will be populated with the SCIPUDO classes to allow clinicians to construct queries, specific to the patient flexibly. All patient data will be stored in a firewall protected secure environment with role-based access control and audit trail logging.

### Development and Validation of the SCIPUD+ Environmental, Social, and Clinical Structural Model

We will develop the SCIPUD+ user interface which will provide a single point of Web-based access to well-annotated and de-identified data generated from multiple domains. Modifiable and nonmodifiable factors will be considered (Figure 1). To develop the SCIPUD+ environmental, social and clinical PrI risk structural model we will consider PrI status as the response variable. We will employ general logistic and multinomial logistic models with linear mixed effects (transformed if necessary) and interaction terms to fit the data. Tree-based models such as classification and regression tree (CART) and Random Forest will also be used to examine the relationship of the factors to the PrI status. Model and variable selection will be implemented to define the SCIPUD+ environmental, social, and clinical model. Final models will be validated using cross-validation. Both models, especially the tree-based models are useful to rank-order factors to identify specific critical variables for an individual, with a focus on modifiable factors (Figure 2).

### Development of the Integrated SCIPUD+ Model

General logistic and multinomial logistic models with linear (mixed) effects and their possible interaction terms will then be fit to the data or their natural groups, and the significance of PrI development will be assessed. Natural groupings will be obtained using a cluster analysis of 5000 medical records and 60 detailed tissue health profiles to examine the association of these natural grouping with PrI frequency. The representativeness of the tissue health group will be compared with the larger sample drawn from the larger cohort extracted from VINCI EHR. The integrated model will be developed in the same way as the SCIPUD+ environmental, social and clinical structural model. Using statistical software R and Splus, tree-based models such as CART and Random Forest, will also be used to examine the relationship of all factors with PrI status. Model and variable selection based on both logistic and tree-based models will be implemented to define the integrated SCIPUD+ model. Final models will be validated using 10-fold cross-validation and some hold out cases using predictive measures. Both models, especially the tree-based models are useful to rank-order factors for identification of specific critical variables for an individual. We shall pay particular attention to modifiable factors. The comprehensive model proposed will allow us to borrow the degrees of freedom from all data points to develop the fully integrated SCIPUD+ Resource. We will determine structural models based on both data sources using standard statistical models, and directly using large-p small-n modern techniques for all factors. Special interest models, such as those focused on modifiable factors, will also be developed. The choice of essential features will depend on the optimization criteria used by a model fitting or learning algorithm. For example, the Random Forest provides 2 criteria for ranking the important features, also known as the variable of importance. One is based on the contribution to Model Accuracy and the other to Gini impurity by each included variable [52-54]. We will use the domain knowledge to guide our final choice of the model for different medical purposes if the final models differ significantly based on various criteria. We may also use XGboost as needed to derive and validate the best predictive model [55]. We expect that at most 25 top-ranked factors will be enough for modeling PrI risk as either a dichotomous or polytomous outcome. This will allow development of the SCIPUD+ care planning algorithm.

## Results

As a preliminary high-level review, we ran a query using the Elixhauser Comorbidity index, which is a tool applied to analysis outcomes of interest to hospital administrators, such as predicting hospital resource use [56,57]. The 30 variables included are all dichotomous. This means that they are either present or absent, which makes categorization much more straightforward than a continuous variable such as the level of injury or even living status. The first outcome is that only 6.00% of the cohort of 40,128 Veterans with SCI have no comorbidities. We also know that many individuals in the cohort have more than one comorbidity. Based on the 5 most commonly coded comorbidities, it was determined that paralysis, the most common at 15.97%, was remarkably low for a cohort of Veterans with SCI. This finding provides an indicator that valuable clinical information is not coded and must be extracted from the clinical notes. The second most common comorbidity is depression. This has also been found in our relational analysis to occur concurrently with many other risk factors. Although we cannot determine which is the cause and which is the effect at this point, we can see it is a major psychological risk factor which will impact many aspects of personalized PrI prevention planning.

To determine the incidence of comorbidities of interest in our cohort, we have identified 226 ICD-9-CM codes of interest. We ran a Structured Query Language (SQL) query across all tables and created a summary table of all comorbidity ICD-9-CM codes. This table contains 1,681,050 records for 32,398 individuals (this total number of individuals varies from the overall cohort total because not all individuals have a recorded comorbidity). The current data represent raw counts which have not been corrected for repeated reports, which may be either a chronic condition such as diabetes or repeated occurrences such as PrIs (Table 1). These extracted data were imported into the query interface adapted from the Physio-MIMI and OnWARD, which enables interactive CDE query and cohort identifications.

**Table 1:** Elixhauser Comorbidity Index Query based on the International Classification of Diseases, Ninth Revision, Clinical Modification (ICD-9-CM) codes for the study cohort (N=32,398).

Parameter		n (%)
**Five most commonly coded comorbidities**		
	Paralysis	6408 (5.97)
	Depression	5324 (13.27)
	Hypertension	4551 (11.34)
	Heart disease	3702 (9.23)
	Substance abuse	3494 (8.71)
**Five least commonly coded comorbidities**		
	Obesity	1452 (3.62)
	Neuro disorders	1125 (2.80)
	Anemia	757 (1.89)
	Weight loss	753 (1.89)
	Gastric disease	230 (0.57)

## Discussion

The project is using established EHR data to build a comprehensive, structured model of environmental, social and clinical PrI risk factors. A concurrent cross-sectional study will develop a structured model of tissue health PrI risk factors. Data from multiple domains will be integrated to provide personalized PrI care planning based on an individual’s risk factor characteristics. The comprehensive SCIPUD+ health care tool will be used to relate the primary outcome of interest (ie, PrI development) with covariates that include environmental, social, clinical, personal and tissue health profiles as well as possible interactions among these covariates. The SCIPUD+ Resource will provide an extremely valuable PrI prevention care planning resource for nurses and other clinical care providers.

The study will result in a validated tool for personalized implementation of CPG recommendations. Maintenance of tissue health provides a foundation for all active duty military and Veterans with SCI to maximize their quality of active life. Recognizing that every person with SCI is an individual; the SCIPUD+ Resource will contribute to Personalized, Proactive, and Patient-Driven care for all. PrI risk characteristics will be used for the development of personalized CPG priority-based care plans for primary and secondary PrI prevention. The use of SCIPUD+ care planning will impact individual health and QoL. Recognizing that health care budgets are limited, the SCIPUD+ Resource will also support optimization of resource capital allocation.

The SCIPUD+ Resource has great potential to change the standard of care for PrI clinical practice by enabling clinicians to provide a personalized application of CPG priorities tailored to the needs of everyone with SCI. The use of our tool will allow clinicians to develop effective personalized care plans for primary and secondary PrI prevention for patients in their care. In the longer term, this research has excellent potential to directly impact standard of care by targeting interventions that will most effectively decrease PrI development for everyone. The population will benefit from a lower PrI incidence, more effective use of resources, and reduced health care costs.

## Abbreviations

AIS: American Spinal Injury Association Impairment Scale
ALS: amyotrophic lateral sclerosis
CART: classification and regression tree
CDE: common data elements
CPG: clinical practice guideline
DTI: deep tissue injury
EHR: electronic health record
ICD-9-CM: International Classification of Diseases, Ninth Revision, Clinical Modification
ICD-10-CM: International Classification of Diseases, Tenth Revision, Clinical Modification
MS: multiple sclerosis
NINDS CDE: National Institute of Neurological Disorders and Stroke Common Data Elements
NLP: natural language processing
OnWARD: Ontology-driven Web-based Research Data Capture
Physio-MIMI: Multi-Modality, Multi-Resource Information Integration Environment for Multi-center Physiological and Clinical Research Studies
PrI: pressure injuries
PU: pressure ulcer
QoL: quality of life
SCI: spinal cord injury
SCIPUD+: Spinal Cord Injury Pressure Ulcer and Deep Tissue Injury
SCIPUDO: Spinal Cord Injury Pressure Ulcer and Deep Tissue Injury Ontology
VINCI: Veterans Affairs Informatics and Computing Infrastructure Database
VHA: Veterans Health Administration
